# COVID-19 Risk Perceptions and Health Behaviors in Puerto Rico

**DOI:** 10.4269/ajtmh.22-0186

**Published:** 2022-07-11

**Authors:** Charlie Vidal, Page D. Dobbs, Emily Herrmann, Daniela Ameijeiras Mena, Ches Jones

**Affiliations:** ^1^ Puerto Rico Public Health Association, Utuado, Puerto Rico;; ^2^ Department of Health, Human Performance and Recreation, University of Arkansas, Fayetteville, Arkansas

## Abstract

Preventative health behaviors were encouraged for all at the beginning of the COVID-19 pandemic. However, as the pandemic continued after 2020, some people stopped implementing all measures. It is unknown if people living in Puerto Rico continued to perform preventive health behaviors throughout the pandemic. The purpose of this study was to explore if the risk perceptions of COVID-19 were associated with preventative health behaviors among Puerto Ricans during the COVID-19 pandemic. A sample from Puerto Rico (*N* = 285) was recruited from January to March 2021 to participate in a cross-sectional, online survey about health behaviors and risk perceptions of COVID-19. Demographics were reported, and a multivariate logistic regression explored the relationships between health behaviors (e.g., handwashing, staying at home, and not allowing visitors) and fear of COVID-19 (outcome variable) and risk of becoming infected with COVID-19 (outcome variable). Those who reported that they washed their hands more often than usual were more likely (adjusted odds ratios = 6.93) to indicate that they were afraid of COVID-19 compared with those who were not performing this behavior. Also, those who reported not leaving their home as much and who did not receive visitors into their house as much as they did before the pandemic were 2.49 and 2.89 times as likely to report being afraid of the virus, respectively, as their counterparts. Although fear may not effectively change all behaviors, it may encourage Puerto Rican adults to practice healthy behaviors that will prevent the spread of COVID-19.

## INTRODUCTION

The WHO declared the coronavirus disease 2019 (COVID-19) as a global pandemic on March 11, 2020.^1^ In response, different countries worldwide issued national states of emergency, and governments enacted health protocols to halt or reduce the spread of the disease. Generally, people were advised to stay at home, large gatherings were banned, schools and nonessential businesses were closed, and quarantine protocols were implemented. On March 30, Wanda Vazquez, Puerto Rico’s governor, imposed one of the most widespread restrictions seen in the United States including a full lockdown on the island, which included schools, government agencies, and all public places except those related to supply food chain, medical, and other businesses deemed necessary (e.g., gas, financial institutions, and eldercare).^2^ Similar to other isolated communities, this created a need for specific communication about the risk of COVID-19 to help promote the uptake of preventative health behaviors.^3^

The WHO has released multiple advisories to the public on simple health strategies to avoid contracting the disease. These include frequent handwashing; avoiding touching eyes, nose, and mouth; social distancing; avoiding gatherings and time spent in crowded public areas; avoiding close contact with someone who is sick; and disinfecting of frequently touched objects and areas.^4^ To help educate the public, primary care physicians, and many within the medical community became the face and voice of public health by providing information about communicable disease prevention strategies to their patients.

With over 45 million COVID cases and 740,000 deaths, the United States has been the epicenter for COVID-19 throughout this pandemic.^5^ Within the United States, health disparities have emerged among particular populations, such as Latino Americans, who have been found to have higher morbidity and mortality rates than non-Hispanic whites.^6^ Different areas of the United States are home to large populations of Latino Americans, including Puerto Rico, an unincorporated territory of the United States. COVID-19 cases were first reported between March 9 and 13, 2020, in Puerto Rico, and as of April 6, 2022, there have been 270,485 confirmed cases and 4,171 deaths attributed to the virus.^7^

The COVID-19 pandemic significantly disrupted and challenged Puerto Rico’s traditional structure of medical care. Further, family practice in Puerto Rico has been challenged and seriously affected because of the strict lockdowns, curfews, and Puerto Ricans’ fear of being exposed and contracting the virus. Studies specific to Puerto Rico indicated that prevention practices and policies (e.g., curfews, quarantines, screenings, and school/business closing enforced by the local government) implemented early during the pandemic likely slowed the spread among targeted populations such as detainees in correctional facilities.^8^^,^^9^ However, there is a gap in research about what Puerto Ricans knew about prevention practices (e.g., washing hands, wearing a mask, and leaving one’s house) after the initial stages of the pandemic. Such information is essential because it can help primary care physicians and those working to create media campaigns to understand and adequately educate Puerto Ricans about the risk of COVID-19 and health behaviors that can help to reduce the spread of future communicable diseases. Thus, the purpose of this study is to explore the risk perceptions of those living in Puerto Rico during the COVID-19 pandemic and to explore health behaviors associated with the perceived risk of the virus.

## MATERIALS AND METHODS

Data were collected from an anonymous, online, cross-sectional survey using Qualtrics between January 26 and March 17, 2021, by a research team that included academic researchers and a primary care physician living in Puerto Rico (the principal investigator). Eligibility included current residence in Puerto Rico and being at least 18 years of age. Participants were recruited from social media networks (e.g., Facebook, Twitter, and Reddit). To do this, the principal author shared the link to the study via social media, and members of the research team shared the link to the survey on prominent COVID-related social media groups targeted at Puerto Rican residents, a data collection strategy used in the authors’ prior research.^10^^,^^11^ Given the physician’s broad following, the study was able to recruit 285 participants. Participants provided informed consent on the first webpage of the survey, and the survey was administered via Qualtrics in both English and Spanish. All study procedures were approved by the University of Arkansas Institutional Review Board (Protocol # 2012305383).

Participants were asked to report demographic information, including age, sex, marital status, educational attainment, race/ethnicity, current chronic diseases, and healthcare workers. Marital status was dichotomized into “married” or “single,” with those who were single, widowed, or divorced included categorized as “single.” Race/ethnicity was dichotomized into “Hispanic/Latino” and “Other”; those identifying as White, Black or African American, Native American or American Indian, Asian/Pacific Islander, and Other were categorized as “Other” due to frequencies. For a complete list of demographic items and response options, see Table 1.

**Table 1:** Participant characteristics (*N* = 184)

Variable		*N* (%)
Age (years)	*M*, SD	39.5 (15.9)
	Minimum, Maximum	19, 84
Gender	Female	126 (68.5)
	Male	58 (31.5)
Race	Hispanic or Latino	152 (82.6)
	Other	32 (17.4)
Marriage status	Single	120 (65.2)
	Married	64 (34.8)
Educational attainment	Less than 4-year college degree	42 (22.8)
	4-year degree or higher	142 (77.2)
Healthcare worker*	Yes	41 (22.3)
	No	143 (77.7)
Immediate family member > 65 years	Yes	103 (56.0)
	No	81 (44.0)
Chronic disease	Yes	54 (29.3)
	No	130 (70.7)

To measure health behaviors, participants were asked a series of questions that measured preventative and risk behaviors that could reduce or increase one’s exposure to COVID-19, respectively. These items were developed to measure preventive and risk behaviors performed by oncology patients from 30 cities in Turkey during April 2020 of the COVID-19 pandemic.^12^ These items included questions about washing hands, wearing a mask, leaving one’s house, and more. For a complete list of behavior items, see Table 2. Participants were also asked about perceived fear of COVID-19 and the risk of contracting COVID-19, items that were originally used in a cross-sectional study that explored COVID-19perceptions and vaccine hesitancy.^13^ These items included: “Are you afraid of the new COVID-19?” and “Do you feel at risk of becoming infected with the new COVID-19?” Responses were measured using a dichotomous yes/no response.

**Table 2:** Preventive and risk health behaviors during COVID-19 pandemic (*N* = 184)

Survey items	Response	*N*	%
Do you wash your hands with soap more often than usual due to COVID-19 pandemic?	Yes	167	90.8
	No	17	9.2
When you leave the house, is it for the hospital, work, school, or just to go out for fun?	Hospital	45	24.5
	Job	106	57.6
	School	26	14.1
	Distraction	75	40.8
Select the products you have purchased due to the COVID-19 pandemic*	Gel sanitizer	168	91.3
	Alcohol	171	92.9
	Disinfectants	156	84.8
	Gloves	98	53.3
When you go out, do you carry hand sanitizer with you?	Yes	158	85.9
	No	3	1.6
	Sometimes	23	12.5
Do you wear a mask every time you leave the house?	Yes	182	99.0
	No	1	0.5
	Sometimes	1	0.5
What is your mask made of?	Cloth mask	31	16.8
	Paper mask	6	3.3
	Surgical mask	124	67.4
	N95	23	12.5
Do you wear gloves when you go out?	Yes	12	6.5
	No	172	93.5
Do you clean the boxes of food and drink you bought at the supermarket when you get home?*	Yes	98	53.3
	No	86	46.7
Are you still leaving your home as usual?	Yes	46	25.0
	No	138	75.0
Are you still receiving visitors in your home?	Yes	65	35.3
	No	119	64.7
Which of the following activities do you participate in as usual?*	Church	36	19.6
	Family activities	57	31.0
	Outdoor	68	37.0
	Gyms	20	10.9
	Other	40	21.7
Are you afraid of the new COVID-19?	Yes	132	71.7
	No	52	28.3
Do you feel at risk of becoming infected with the new COVID-19?	Yes	125	67.9
	No	59	32.1

* Participants could select more than one option.

Based on a G*Power analysis,^14^ the recommended sample size for a logistic regression with the effect size of 1.2 would be 91 participants. Of the 285 participants recruited to complete the survey, 17 did not meet eligibility criteria, 63 did not provide consent to participate or did not continue to complete the survey after reading the consent page, and 21 did not complete relevant variables for the analyses. Thus, after cleaning all data, 184 participant responses remained for analysis, which was determined to be sufficient for the current analyses. After reporting descriptive statics, logistic regressions were used to examine associations between independent variables (i.e., demographics variables, health behaviors) and perceived fear of COVID-19 (outcome/dependent variable) and perceived risk of contracting COVID-19 (outcome/dependent variables). Odds ratios (OR) and adjusted odds ratios (aOR) were reported along with 95% CI. Alphas for all analyses were set a priori to 0.05. Variability was reported using Nagelkerke *R*^2^.

## RESULTS

Of the 184 participants, most identified as female (*N* = 126, 68.5%), were Hispanic or Latin (*N* = 152, 82.6%), and were single (*N* = 120, 65.2%). Overall, the mean age of participants was 39.5 years (SD = 15.9, ranging from 19 to 84), and 77.4% (*N* = 142) had completed a 4-year college degree with 41% (*N* = 22.3%), indicating that they were a healthcare worker. Among the sample, 103 (56.0%) participants reported that they lived with someone 65 years of age or older, and 54 (29.3%) responded that they had at least one chronic disease. See Table 1 for full participant characteristics.

The majority of the sample reported that they washed their hands more often than usual (*N* = 167, 90.8%), they wore a mask when they left their house (99.0%), and that they carried hand sanitizer when they did leave their home (*N* = 158, 85.9%). Overall, most people reported that they did not leave their house as usual as before the pandemic (*N* = 138, 75%), with the most commonplace people reporting going when they did leave their house being their job (*N* = 106, 57.6%). Most participants reported that they had purchased gel sanitizer (*N* = 168, 91.3%), alcohol (*N* = 171, 92.9%), disinfectants (*N* = 156, 84.8%), and gloves (*N* = 98, 53.3%); however, most participants did not wear gloves when they were in public (*N* = 172, 93.5%). See Table 2 for participant responses to all health behavior items.

Among our sample, 46 (25.0) participants indicated that they were still leaving their house as usual. Further, 65 (35.3%) participants reported that they were still receiving visitors in their home. Activities in which participants reported engaging included church (*N* = 36, 19.6%), family activities (*N* = 57, 31.0%), outdoor activities (*N* = 68, 37%), gyms (*N* = 20, 10.9%), and other activities (*N* = 40, 21.7%) as usual.

When regressed together, items associated with fear of COVID-19 included washing hands more often than usual, leaving one’s home, and allowing visitors inside one’s home. Those who reported that they washed their hands more often than usual were more likely (aOR = 6.93; 95% CI = 2.00–23.99) to indicate that they were afraid of COVID-19 than those who were not performing this behavior. Those who said they were not leaving their house as they usually would before the pandemic was 2.49 (95% CI = 1.07–5.82) times as likely to report being afraid of the virus as those who were leaving their house as usual. Lastly, those who were not receiving visitors in their home were significantly more likely (aOR = 2.89; 95% CI = 1.26–6.65) than those who allowed others to visit inside their home to report fear of COVID-19 (see Table 3). Although these factors were associated with fear of the virus, no factors were significantly associated with the perceived risk of becoming infected by COVID-19. The regression models were found to explain 28.5% of the variability about being afraid of COVID-19 and 13.3% of the perceptions that one could become infected by the virus.

**Table 3:** Factors associated with Puerto Ricans fear of COVID-19 and perceived risk of becoming infected with COVID-19 (*N* = 184)

	Afraid of COVID-19		Risk of becoming infected	
Variables	aOR	95% CI	aOR	95% CI
Age	1.03	0.99, 1.06	1.02	0.99, 1.04
Sex				
Male	1.00	–	1.00	–
Female	0.81	0.34, 1.92	0.53	0.24, 1.19
Marital status				
Single	1.00	–	1.00	–
Married	1.08	0.44, 2.66	0.87	0.40, 1.92
Educational attainment				
Less than 4-year college degree	1.00	–	1.00	–
4-year college degree or more	0.80	0.31, 2.07	1.98	0.89, 4.40
Race/Ethnicity				
Other (non-Hispanic White–Black)	1.00	–	1.00	–
Hispanic or Latino	1.28	0.46, 3.56	1.76	0.73, 4.23
Chronic disease				
No	1.00	–	1.00	–
Yes	0.78	0.29, 2.08	1.65	0.68, 4.00
Healthcare worker				
No	1.00	–	1.00	–
Yes	0.57	0.24, 1.36	0.78	0.34, 1.77
Household member 65 years or older				
No	1.00	–	1.00	–
Yes	1.66	0.77, 3.57	0.81	0.41, 1.61
Wash your hands with soap more often than usual				
No	1.00	–	1.00	–
Yes	**6.93**	**2.00, 23.99**	2.64	0.87, 7.99
Carry hand sanitizer				
No/Sometimes	1.00	–	1.00	–
Yes	1.34	0.43, 4.20	1.54	0.54, 4.38
Clean boxes from supermarket				
No	1.00	–	1.00	–
Yes	0.86	0.39, 1.91	1.00	0.49, 2.02
Leave house as usual				
No	**2.49**	**1.07, 5.82**	0.93	0.42, 2.09
Yes	1.00	–	1.00	–
Receiving visitors				
No	**2.89**	**1.26, 6.65**	1.60	0.75, 3.41
Yes	1.00	–	1.00	–
*R*^2^	0.285	–	0.133	–

aOR = adjusted odds ratios. Bolded text indicates significance.

## DISCUSSION

We sought to explore the perceived risk of COVID-19 among those living in Puerto Rico and the behaviors associated with such risk. While initial studies about COVID-19 in Puerto Rico were about slowing the spread of the virus^9^ via prevention practices^8^ and about morbidity and mortality risks for those living on the island,^15^^,^^16^ there is a gap in the literature about the prevention practice people living in Puerto Rico are using to protect themselves and their family after the first months of the pandemic. Thus, in the current study, participants answered questionnaires to assess their health behaviors and risk perceptions of COVID-19.

More than 90% of the respondents washed their hands with soap more often than usual due to the pandemic. Also, those afraid of COVID-19 were nearly seven times as likely to report washing their hands with soap more often than usual. This was similar to a sample of 300 oncology patients in Turkey, where more than 97.3% of the participants reported that they washed their hands more often due to COVID-19.^12^ Given that males, those living with a chronic disease, and those who are older are at an elevated greater risk of hospitalization and death than their counterparts,^17^ it seems intuitive that cancer patients would have a heightened awareness of protecting themselves from COVID-19; however, our findings are a promising sign that participants in our study were following health protocols implemented by the authorities at the time data were collected. Some suggest that primary care physicians’ education about responses to COVID-19 (including simple prevention strategies) can help improve health outcomes for their patients.^18^ Thus, those working in family medicine in Puerto Rico can use this opportunity to promote health and prevention of diseases not only for adults but also for children influence the health behaviors of their patients. For example, at times that the vaccine is not available for children younger than 5 years old, useful evidence-based advice from physicians can encourage patients to practice simple prevention strategies.

At the time data were collected, vaccines were only available to the most vulnerable to contracting COVID-19, including healthcare professionals. Thus, stay-on-home orders, social distancing, face masks, and quarantine were strategies advised and/or mandated by local governments to reduce the spread of the disease.^19^ Participants in our study reported only leaving their homes to go to work or participate in a leisure activity. While such mitigation strategies were used to prevent further outbreaks of COVID-19 and reduce the spread of the disease,^20^ it is unknown if all were compliant with such measures, given confusion among some populations.^21^ Further, some behaviors that could reduce the spread of COVID-19 (e.g., mask wearing) were politicized in the United States due to a hostile political climate during the pandemic.^22^ Also, some have argued that the well-being of some marginalized populations and disparate communities suffered from the lack of mental and social engagement.^17^ It is important to note that the least common reason for leaving one’s house was “attending school.” This may have been since all participants were at least 18 years of age, and schools were closed, as implemented by the government, at the time data were collected. However, most participants indicated that they stayed at home and did not accept visitors due to the pandemic.

Health behavior theories, such as the Health Belief Model, have been used to encourage behavior change and communication between healthcare providers and their patients during the COVID-19 pandemic.^23^^,^^24^ This particular theory recognizes the perceived threat of a health outcome, such as contracting COVID-19, through one’s perceived susceptibility and perceived severity of the health outcome.^25^ Among our sample, the perceived susceptibility and severity of contracting COVID-19 appear to be high among our sample, with 67.9% reporting that they believed they were at risk for contracting COVID-19 and 71.7% reporting that they were afraid of the virus. Thus, while scare tactics that increase perceived susceptibility and severity may not be effective for promoting the COVID-19 vaccine among those who are hesitant,^26^ they may be more effective for the promotion of behaviors that will reduce the exposure to and spread of COVID-19. Further, the differences between these two perceptions also highlight the importance of measuring different constructs of behavioral perceptions within the context of COVID-19. While some may fear the virus, they may believe that they have reduced their risk potential for contracting the disease. Alternatively, some may believe they were at risk of contracting the disease; however, they may believe that they would only have mild symptoms and are thus not afraid of the virus. Our findings provide practical advice for those in family practice to encourage their patients to use medication measures to prevent the spread of COVID-19 or other communicable disease in the future. Further, our findings suggest that theoretical behavioral change models can be an effective method to understand and change the behavior of patients.

Limitations included the sampling strategy and the research design. The convenience sampling strategy limits the studies’ external validity or ability to generalize findings to other populations or even those living in Puerto Rico. Further, the cross-sectional nature of the data prohibits the findings from any causal claims, and given that the survey was distributed by a well-known primary care provider who lived in the area, some participant bias may have occurred—with participants attempting to respond in a way that they thought would please their primary care provider. We found our sample to report higher educational attainment (78.3% had a 4-year degree or higher) than the 27.2% of Puerto Ricans who have completed a bachelor’s degree or higher.^27^ Finally, some ORs had wide CIs, and these findings may be interpreted with caution.

Among our sample, we found fear of COVID-19 to be strongly related to health behaviors encouraged during the COVID-19 pandemic, including washing one’s hands with soap more often than usual, not leaving one’s house as usual, and not receiving visitors during the pandemic. While these strategies were essential during the high of this global pandemic. It can be useful for medical teaching hospitals to provide strategies to encourage patients to reduce their risk of infections by health promotion and cultural competence strategies to prevent diseases in their family physician residency programs. With constant changes to the social guidelines provided by the Centers for Disease Control and Prevention and the Worth Health Organization, those working with the public may be asked by their patients when they can resume everyday social interactions with others. With primary care physicians as the face of public health knowledge, they are encouraged to educate their patients about safe behaviors that can reduce health disparities and achieve equitable health. The incorporation of evidence-based health promotion and strategic messages to prevent diseases can have a positive impact on individuals and their broader communities.
